# In Situ Analysis of Oxygen Vacancies and Band Alignment in HfO_2_/TiN Structure for CMOS Applications

**DOI:** 10.1186/s11671-017-2068-y

**Published:** 2017-04-27

**Authors:** Da-Peng Xu, Lin-Jie Yu, Xu-Dong Chen, Lin Chen, Qing-Qing Sun, Hao Zhu, Hong-Liang Lu, Peng Zhou, Shi-Jin Ding, David Wei Zhang

**Affiliations:** State Key Laboratory of ASIC and System, School of Microelectronics, Fudan University, Shanghai, 200433 China

**Keywords:** Oxygen vacancies, Band alignment, In situ XPS, UPS, Ellipsometry

## Abstract

The density of oxygen vacancies characterization in high-k/metal gate is significant for semiconductor device fabrication. In this work, a new approach was demonstrated to detect the density of oxygen vacancies by in situ x-ray photoelectron spectroscopy (XPS) and ultraviolet photoelectron spectroscopy (UPS) measurement. Moreover, the band alignment of the structure with optical band gap measured by spectroscopic ellipsometry (SE) and valence band offset by UPS were reported. The specific areal density of oxygen vacancies in high-k dielectric of HfO_2_/TiN was obtained by fitting the experiment data to be 8.202 × 10^10^cm^− 2^. This study would provide an effective approach to characterize the oxygen vacancies based defects which cause threshold voltage shifts and enormous gate leakage in modern MOSFET devices.

## Background

With the conventional Si-based CMOS technology scaling down, high-k/metal gate are regarded as one of the most important structures in modern MOS devices. Among various types of High-k/metal gate, HfO_2_/TiN structure has been proved to be one of the standards in high-k integration in CMOS technology below 28 nm technical node due to its large band offsets, thermodynamic stability in contact with Si, and excellent high-frequency response [1–6]. However, the oxygen vacancies in HfO_2_ is the main obstacle for practical application: The oxygen vacancies in high-k dielectric would cause Threshold Voltage Shifts (TVS) [7] and enormous Gate leakage [8] which may affect MOSFET performance. Moreover, the presence of oxygen vacancies generate a high concentration of charge traps and scattering centers [9], which caused the degradation of mobility in the channel of MOSFET. Few works studied the determination of the density of oxygen vacancies in the HfO_2_ film through experimental methods. In this work, we use *In-situ* [10] Measurement System to investigate the feature of HfO_2_/TiN Structure manufactured by PVD and ALD, with X-ray Photoelectron Spectroscopy (XPS) and Ultraviolet Photoelectron Spectroscopy (UPS) applied to get the composition of the film and the work functions of TiN metal with different thickness of HfO_2_ dielectric in 0.2, 0.5, 0.8, 1.2, 1.6, 2.0, 2.5 and 3.0 nm. By fitting the curves of different TiN work functions due to the ultraviolet activation by UPS and extracting the fitted parameters, the density of oxygen vacancies in HfO_2_ film can be achieved. Moreover, both the band alignment of the structure with optical band gap measured by spectroscopic ellipsometry (SE) and valence band offset by UPS are also reported in this work.

## Methods

**Fig. 1:**
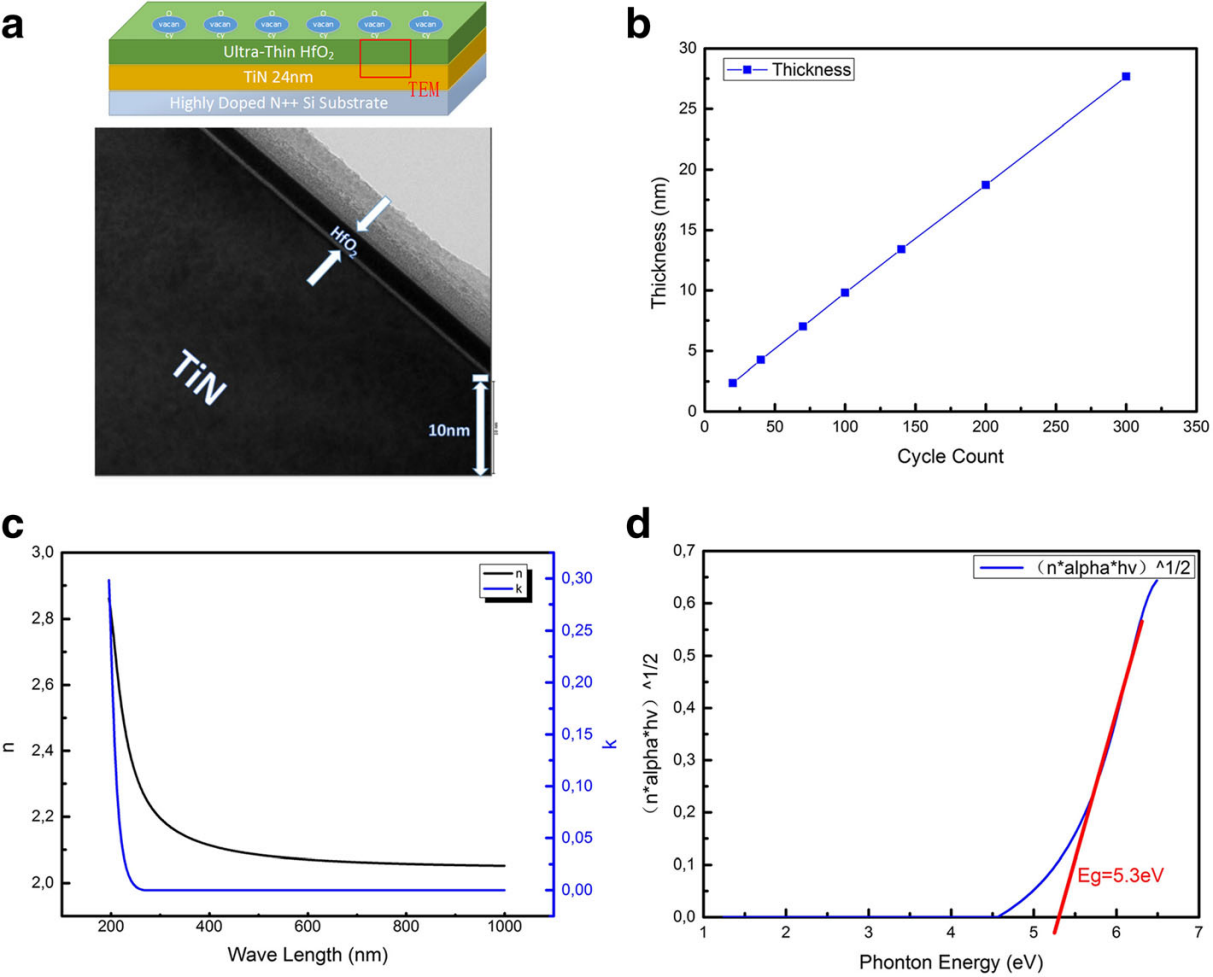
Material characterization. **a** Structure and TEM images of HfO_2_/TiN/Si structure. **b** Growth rate measurement of HfO_2_. **c** Optical constant of HfO_2_. **d** Band gap extraction of HfO_2_

The structure of Fig. 1 (a) has been manufactured by SPECS *In-Situ* Cluster System and Picosun ALD system. Firstly, 24 nm TiN was deposited on highly-doped silicon wafer by SPECS PVD Sputtering for 3000 s with Titanium target and Nitrogen. Highly-doped silicon wafer was used to eliminate the accumulation of the charge effect during UPS measurement caused by negative substrate voltage. Then the original work function of TiN was measured by UPS. Subsequently, 0.2, 0.5, 0.8, 1.2, 1.6, 2.0, 2.5 and 3.0 nm HfO_2_ were deposited on the TiN surface by Picosun ALD with TEMAH precursor (70 °C) and H_2_O at certain reactor temperature (250 °C) [11] in high vacuum chamber. During each thickness of HfO_2_ growing process (0.2, 0.5, 0.8, 1.2, 1.6, 2.0, 2.5 and 3.0 nm), the sample was transferred to the XPS chamber. Then, *in-situ* XPS and UPS were measured with mono-Al target X-ray source and HeI Ultraviolet source utilized in XPS and UPS. Also, we apply -5 V voltage to the substrate holder during UPS measurement for shifting our spectrum position to observe the cutoff edge, respectfully. The manufacture and measurement process were *In-situ* process in high vacuum chambers. The composition of the film, the work function of the TiN and the Valence Band Offset (VBO) of TiN/HfO_2_ were also calculated from the results. The growth rate of ALD-HfO_2_ was measured by J.A.Woollam *In-situ* SE with different thickness of HfO_2_ deposited instantly on Si substrate by Picosun ALD. The optical band gap of HfO_2_ can also be extracted from this measurement. With TiN work function, HfO_2_/TiN VBO and optical band gap of HfO_2_ (E_g_), the band alignment of HfO_2_/TiN has been studied in this paper. Moreover, oxygen vacancies can be calculated from the change of the TiN work function caused by the ultraviolet stimulus of oxygen vacancies in HfO_2_ layer by UPS during the growth of HfO_2_.

## Results and Discussion

The structure and TEM images of HfO_2_/TiN/Si has been shown in Fig. 1(a). The structure of our test chip is a sandwich structure with HfO_2_, TiN and silicon substrate. The thickness of HfO_2_ and TiN is 3 nm and 24 nm after the whole process. The ALD growth rate, the optical constant and the band gap of HfO_2_ has been presented in Fig. 1(b), (c) and (d) which were measured by *In-situ* SE. From Fig. 1(b), 20, 40, 70, 100, 140, 200, 300 cycles HfO_2_ were deposited directly on the clean 4-inch silicon wafer substrate by ALD process to measure the growth rate of HfO_2_. The thickness of 20, 40, 70, 100, 140, 200, 300 cycles HfO_2_ were measured to be 2.36, 4.25, 7.01, 9.80, 13.42, 18.73 and 27.67 nm. From the slope of the line, the growth rate of HfO_2_ for the experiment is 0.09 nm/cycle which is reasonable in ALD deposition [11]. Moreover, the optical constant of the film is presented in Fig. 1(c). From the Figure, the index of refraction (n) of the HfO_2_ film decrease with the increasing of λ. The index of refraction (n) of the HfO_2_ film at 632 nm is 2.07 which is slightly larger than the standard 1.9 due to the defect of the film but in good agreement with Liu et al. [12]. The k value of the HfO_2_ is not zero in low λ which indicates the absorption of the film, which can simultaneously indicate the defects in HfO_2_. The optical bandgap of the HfO_2_ was 5.3 eV in our work determined by plotting the empirical expression ${\left(\mathrm{n}\upalpha \mathrm{h}\upgamma \right)}^{\frac{1}{2}}$ versus hγ, as shown in Fig. 1(d), where n, α and hγ is the index of refraction, the absorption coefficient and the photon energy as described in Yang et al. [13]. The extracted E_g_ value (5.3 eV) is in good agreement with previously reported values from 5.25 eV to 5.8 eV for HfO_2_ [14–16] in different references.

**Fig. 2:**
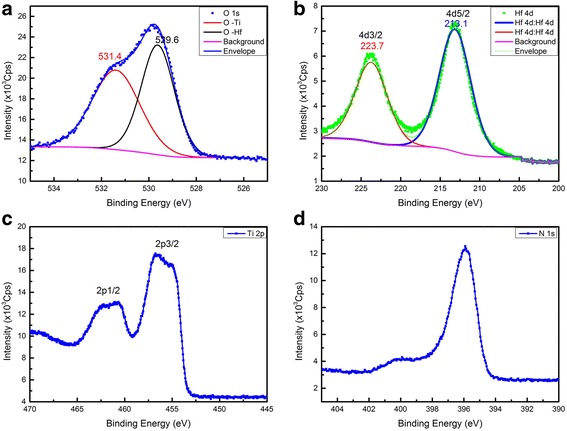
XPS of HfO_2_/TiN structure. **a** O 1s spectrum. **b** Hf 4d spectrum. **c** Ti 2p spectrum. **d** N 1s spectrum

**Fig. 3:**
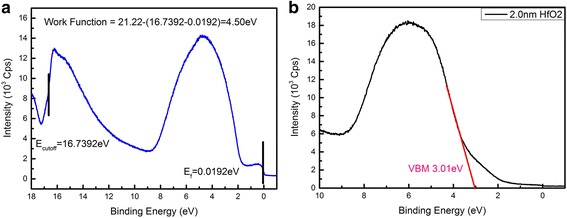
UPS measurement. **a** UPS of TiN. **b** UPS of 2-nm HfO_2_ on TiN

**Fig. 4:**
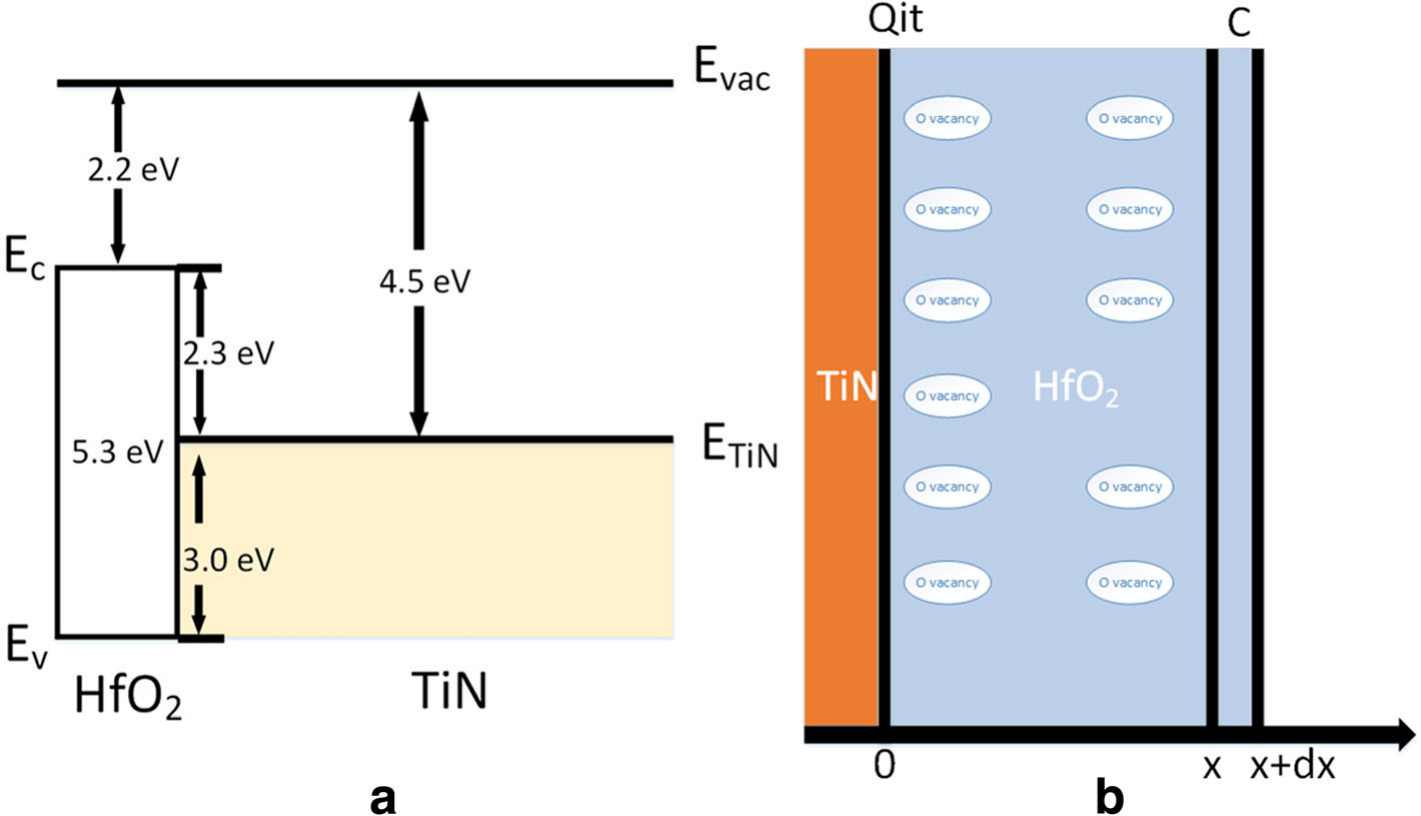
Band alignment and structure. **a** Band alignment of HfO_2_/TiN structure was measured by the UPS and SE measurement. **b** Structure of TiN/HfO_2_ capacitor was used to introduce the calculation method of the areal density of the oxygen vacancies

The XPS diagram of HfO_2_ (2 nm)/TiN structure was shown in Fig. 2 after calibrated by C 1 s Binding Energy (284.6 eV).The elemental stoichiometry Hf : O was calculated to be 1:1.96 from the XPS data. Because of the limit of the measurement depth of X-ray source, there were only five kinds of element (Ti, N, O, Hf and C), which is presented in Fig. 2 (except the Carbon). Two components were fitted to the O1s spectrum in Fig. 2(a) for 531.4 eV (Ti-O) and 529.6 eV (Hf-O) and there were two peaks of Hf 4d diagram because of the energy level splitting of 4d3 and 4d5 in Fig. 2(b). In Fig. 2(c), Ti was not accurate enough to fit for the fact that the TiN film actually is the mixture of Ti_2_N_2_, Ti_3_N_4_, TiON interface and some other their compounds which also was covered by a layer of HfO_2_. Fig. 2(d) is the XPS diagram of N 1 s which can prove the existence of TiN layer. The work function of the TiN was measured to be 4.50 eV in Fig. 3(a) by UPS, which was calculated by the energy of HeI (21.22 eV) ultraviolet, the Fermi Edge (0.02 eV) and the Cut-off Edge (16.74 eV) of UPS diagram. The Valence Band Offset (VBO) was measured to be 3.01 eV by the UPS of 2.0 nm HfO_2_ deposited on TiN in Fig. 3(b). With TiN work function (4.5 eV), HfO_2_/TiN VBO (3.0 eV) and optical band gap of HfO_2_ (5.3 eV), the band alignment of HfO_2_/TiN has been reported in Fig. 4. The Conduction Band Offset (CBO) is 2.3 eV as presented in Fig. 4(a).The results are reasonable compared to calculation result 3.0 eV [17] using WM method based on ab initio calculations.

**Fig. 5:**
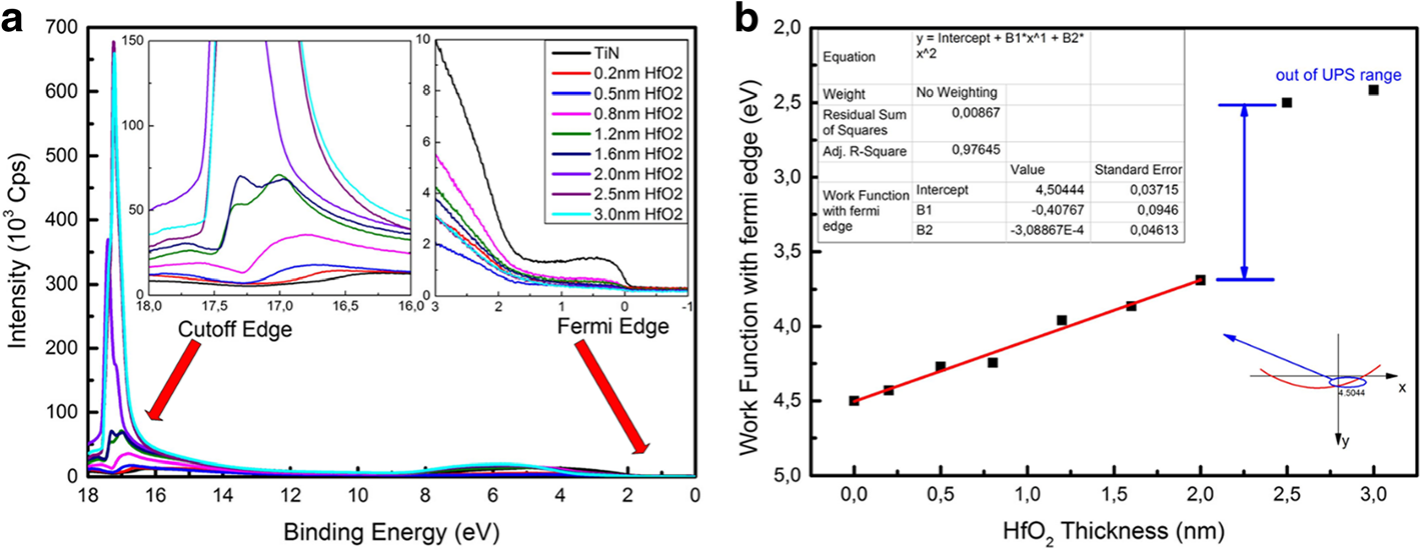
Extraction of density of oxygen vacancies. **a** UPS of HfO_2_/TiN structure. The cut-off edge has been normalized to [0, 1]. **b** Oxygen vacancies fitting result from work functions of TiN

The UPS spectrum of 0.2, 0.5, 0.8, 1.2, 1.6, 2.0, 2.5 and 3.0 nm of HfO_2_ deposited on the TiN has been given in Fig. 5(a). The Fermi Edge of the UPS is measured by the point of inflection of the curve and the Cut-off Edge of the UPS is chosen by the middle point of the maximum and the minimum. The Fermi Edge is around zero point due to the charge effect, and the Cut-off Edge is shifted to the direction of high binding energy which indicates the decrease of the TiN work function with increasing HfO_2_ thickness from Fig. 5(a). The difference of the work function was caused by the oxygen vacancies activation with HeI UV source applied during the UPS measurement. The work function of the 2.5 nm and 3 nm HfO_2_ is not correct due to the charge effect caused by the limited depth of UPS. The gap between 2.0 and 2.5 nm in Fig. 5(b) and the nearly same curve of the UPS spectrum in Fig. 5(a) indicates that those two points should beyond the range of UPS depth.

The density of oxygen vacancies was extracted from the experiment data with the simple capacitor physics equation. Fig. 4(b) is the TiN/HfO_2_ structure from left to right. In Fig. 4(b), take x to x + dx as a parallel capacitor. According to the basic charge equation,

1 $$ \mathrm{Q}=\mathrm{C}\times \mathrm{V} $$

Where Q is the charge, C is the capacitor and V is the voltage between the capacitor.

Considering the activated interface trap in HfO_2_/TiN interface and the oxygen vacancies density in body, we got

2 $$ {\mathrm{Q}}_{it}+{\delta}_x x S=\frac{\varepsilon_{HfO2} S}{dx} d V $$

Where Q_*it*_ is the charge of HfO_2_/TiN interface trap, *δ*_*x*_ is the bulk charge density of the oxygen vacancies, *S* is the area of the wafer and *ε*_*HfO*2_ is the dielectric constant (the relative dielectric constant is 16, which is calculated by SE data and verified by C-V measurement, and the *ε*_0_ is 8.85 × 10^-12^ F/m).

After integration, then note ${\mathrm{q}}_{it}\equiv \frac{Q_{it}}{S}$, which is the Areal density of the charge of interface trap. The change of the work function in UPS diagram in Fig. 5(b) is caused by the charge of oxygen vacancies. Therefore,

3 $$ \mathrm{V}\left(\mathrm{x}\right)=\frac{{\mathrm{W}}_F\left(\mathrm{x}\right)}{e}=\frac{\delta_x}{2{\varepsilon}_{HfO2}}{x}^2+\frac{q_{it}}{\varepsilon_{HfO2}} x+ A $$

Where e is the charge of the electron for 1.6 × 10^-19^C.

After the TiN work function fitting process in Fig. 5(b) by quadratic function, we got $\frac{\delta_x\times e}{2{\varepsilon}_{HfO2}}=3.089\times {10}^{14}\ \left( V\cdot \frac{C}{m^2}\right)$ and $\frac{q_{it}\times e}{\varepsilon_{HfO2}}=4.077\times {10}^8\ \left( V\cdot \frac{C}{m}\right)$. After the calculation, the bulk charge density of the oxygen vacancies is *δ*_*x*_ = 5.468 × 10^17^ *cm* ^− 3^. The oxygen vacancies in HfO_2_ may exist in five charge states, +2, +1, 0, −1, −2 corresponding to up to four extra electrons in the vicinity of the vacant O^2−^ site [9]. However, the Ultraviolet source from UPS measurement will activate the electron in defects and make the oxygen vacancies to be +2 states. After changed to areal density by multiply the HfO_2_ thickness (2 nm) and divided by 2 (one oxygen vacancy has two charges), the areal density of the oxygen vacancies is 8.202 × 10^10^ *cm* ^− 2^ and the areal density of the interface trap is *q*_*it*_ = 3.608 × 10^13^ *cm* ^− 2^.This result is acceptable because the sample was not annealed in forming gas which would make *q*_*it*_ larger.

## Conclusions

In summary, we successfully realized the measurement of the band alignment and the determination of the density of oxygen vacancies by *in-situ* SE, XPS and UPS with different thickness of HfO_2_ dielectric in 0.2, 0.5, 0.8, 1.2, 1.6, 2.0, 2.5 and 3.0 nm on TiN metal layer. The density of oxygen vacancies was extracted from the experiment data with the simple capacitor physics equation. In TiN/HfO_2_ Band Alignment, the work function of the HfO_2_ optical band gap is 5.3 eV,and the VBO and CBO of HfO_2_/TiN is 3.0 eV and 2.3 eV. The areal density of the oxygen vacancies is calculated to be 8.202 × 10^10^ *cm* ^− 2^. This method provides a direct way to capture the density of oxygen vacancies of the high-k/metal-gate for advanced semiconductor MOSFETs.

## Abbreviations

SE: Spectroscopic ellipsometry
TVS: Threshold voltage shifts
UPS: Ultraviolet photoelectron spectroscopy
VBO: Valence band offset
XPS: X-ray photoelectron spectroscopy
